# Prediction of Deflection of Reinforced Concrete Beams Considering Shear Effect

**DOI:** 10.3390/ma14216684

**Published:** 2021-11-05

**Authors:** Sang-Woo Kim, Kil-Hee Kim

**Affiliations:** 1 Department of Civil, Architectural and Environmental System Engineering Graduate School, Sungkyunkwan University, Suwon 16419, Korea; swkim91@skku.edu; 2 Department of Architectural Engineering & Urban System Engineering, Kongju National University, Cheonan 31080, Korea

**Keywords:** shear effect, deflection, effective moment of inertia, flexure, RC beams

## Abstract

This paper proposes a method to evaluate the effect of shear on the deflection of reinforced concrete (RC) beams. The deflection of RC beams due to the effects of flexural and shear cracks shows different results from those obtained from the elastic theory. The effect of shear on deflection was compared and analyzed in this study, on the basis of experimental results and elastic theory using the virtual work method. The shear effect on the deflection of RC beams by elastic theory was extremely small. However, experimental results showed a difference of over 40% from the results predicted by elasticity theory. In this study, a new method was developed to reasonably predict the deflection of flexure-critical RC beams using the deflection incremental coefficient due to shear. The proposed method was compared with the existing experimental results obtained from the literature for verification. As a result of the comparison, the deflection obtained using ACI 318-19 underestimated the actual deflection by approximately 33%, whereas the deflection obtained by the proposed method predicted the experimental results relatively accurately.

## 1. Introduction

Many structural codes consider the deflection of structures in terms of safety and serviceability. Safety ensures that no casualties result from structural collapse. For this, it is necessary to provide sufficient time for people to identify deformations, such as deflection of the structure and to evacuate before destruction. Meanwhile, the serviceability of a structure is intended to avoid inconvenience to users, and the structural standards limit it to prevent excessive vibration or deflection. For safety purposes, it is conservative for the analytical result of deflection in the ultimate state to be smaller than the actual deflection of structural members. In contrast, for the purpose of serviceability, it is conservative for the analytical result to be larger than the actual deflection of structural members in a service load.

The actual deflection of structural members using non-cracking materials, such as steel structures, is not significantly different from the deflection calculated based on the general elastic theory. However, in the case of using a material that can crack even under a service load, such as reinforced concrete (RC) structures, the flexural stiffness and deflection are different from those predicted by elastic theory because of the effect of cracking. 

In 1965, Branson [1] proposed an equation for the effective moment of inertia of RC beams using cracking and yield moments. The Branson equation was reflected in the ACI building code from 1971 [2] to 2018 [3] because the deflection of the structural member can be easily calculated by substituting it into the elastic deflection equations. Bischoff [4,5] reported that the Branson equation deviated significantly from the experimental results for the deflection of RC beams with a tensile reinforcement ratio of less than 1%. Scanlon and Bischoff [6] studied the effect of shrinkage restraint cracking and loading history, and ACI 318-19 [7] modified the equation for the effective moment of inertia based on the research results reported by Bischoff et al. 

**Figure 1:**
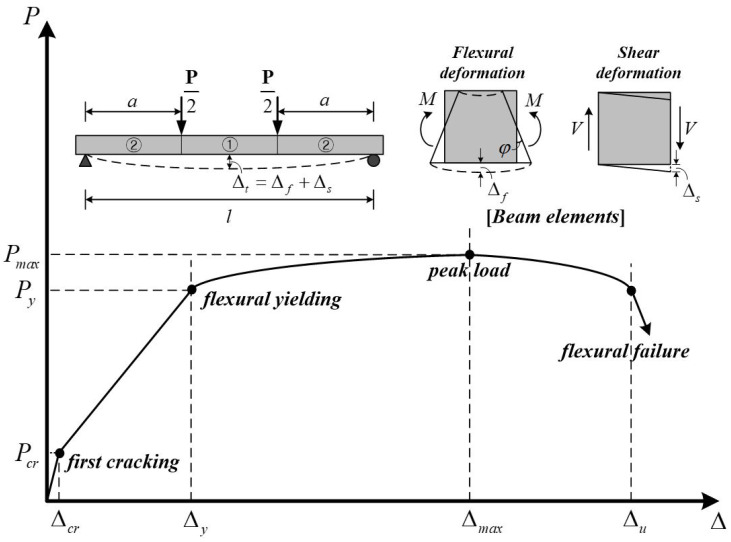
Typical load-deflection behavior of flexure-critical RC beams.

Figure 1 shows the typical load-deflection behavior of flexure-critical RC beams that failed in flexure. As shown in Figure 1, the beam members are generally subjected to bending and shear. In the pure bending region ①, only flexural deformation occurs, whereas in region ② where both flexure and shear exist, both flexural and shear deformations occur. After cracking by an external load, the RC members exhibit a different behavior from that described by the elastic theory. Several researchers, including Branson and Bischoff, have conducted studies to evaluate the effect of cracks on flexural stiffness. Similarly, when a shear crack occurs in an RC beam, the deformation is concentrated in the crack, showing different characteristics from the deformation according to the general elastic theory. However, most studies on deflection have focused on flexure based on elastic theory, which implies that shear does not have a significant effect. 

Recently, Kim et al. [8] experimentally separated and evaluated the effects of flexure and shear on deflection by conducting flexural tests of simply supported flexure-critical RC beams subjected to concentrated loads. Their results indicated that the deflection calculated using ACI 318-19 was similar to the deflection caused by flexure. Furthermore, it was reported that the total deflection reached a maximum of approximately 1.6 times the measured pure flexural deflection. In this study, a new method is developed for a more accurate evaluation of the deflection of flexure-critical RC beams considering the shear effect. 

## 2. Review of Previous Study

### 2.1. Effect of Shear on Deflection of Flexure-Critical RC Beams 

**Figure 2:**
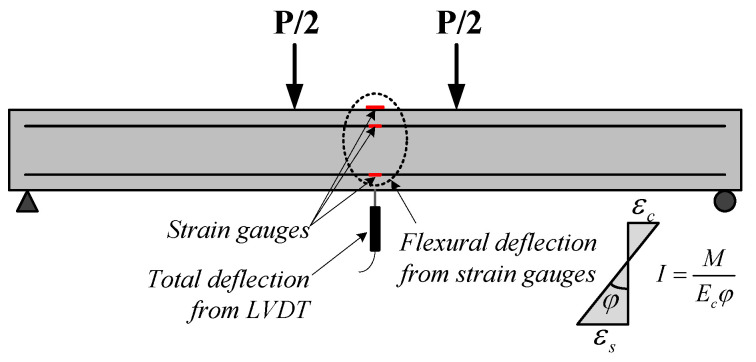
Flexural and total deflections of an RC beam.

Kim et al. [8] experimentally evaluated the effect of shear on the deflection of flexure-critical RC beams. As shown in Figure 2, the deflection at the mid-span of the beam can be obtained using (1) a linear variable differential transducer (LVDT) and (2) strain gauges attached to the mid-span of the beam. The values measured from the strain gauges were used to evaluate the deflection due to flexure (flexural deflection) using the moment-curvature relationship and elastic deflection formula, as detailed in Section 3.2. Because the deflection of RC beams is affected by flexure and shear, the deflection due to shear (shear deflection) can be obtained by subtracting the flexural deflection from the total deflection. 

The experimental results showed that the shear and tension steel bars had no significant effect on the shear deflection of flexure-critical RC beams when the shear reinforcement satisfied the ACI building code. However, it was confirmed that the effect of shear on deflection increased as the shear span-to-depth ratio decreased. In addition, the deviation between the deflection calculated using the current ACI code [7] and the experimental deflection measured from LVDT increased as the shear-to-depth ratio decreased, and the actual deflection was also underestimated. In this study, a new method to predict the deflection of flexure-critical RC beams is proposed based on the results of a previous study [8,9] that experimentally evaluated the effect of shear on the deflection of RC beams. 

### 2.2. Experimental Program 

Table 1 shows the details and results of the specimens tested in the previous study [8,9]. The test variables were the shear span-to-depth ratio and shear capacity ratio $V_{shear}/V_{flexure}$. It should be noted that the shear reinforcement had no effect on the shear deflection when the shear capacity was greater than the flexural capacity. The specimens classified into three groups according to the shear span-to-depth ratio: 2.5, 3.0, and 4.0. In the name of the specimens, B6 indicates that the tension reinforcement ratio is 60% of the balanced steel ratio for the singly reinforced section. 

Type I Portland cement, blast furnace slag powder, and fly ash were used to prepare ready-mixed concrete. The blast furnace slag powder and fly ash constituted 15.2% and 17.9%, respectively, of the total binder. A water binder ratio of 53.6% and an air-entraining agent, which formed 0.6% of the total binder, were used to obtain the target strength and workability of the concrete. The slump of the concrete was 150 mm. The crushed limestone used as coarse aggregates had a maximum size of 25 mm and a specific gravity of 2.61. The washed sand used as fine aggregates had a specific gravity of 2.59. 

The average compressive strength and the elastic modulus of the concrete was 26.8 MPa and 1.7 × 10^4^ MPa, respectively. In addition, an average of 4.1 MPa was obtained as a result of the test of the modulus of rupture according to ASTM C78 [10]. D22 (387.1 mm^2^) was used for tension reinforcement, and the strength and strain at yield were 543.4MPa and 0.00304, respectively, as a result of the tension test. For compression and shear reinforcement, D16 and D10 were respectively used, and the respective yield strengths were 546.5 MPa and 322.0 MPa.

**Figure 3:**
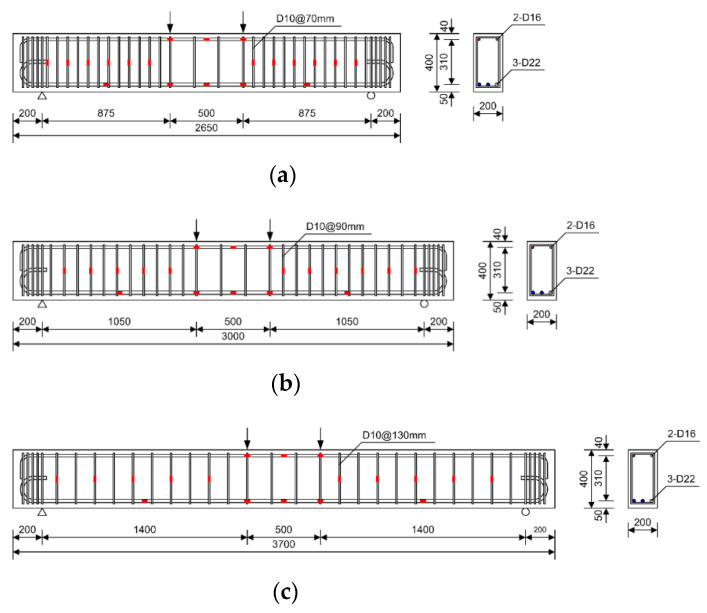
Details of typical specimens [8,9]: (**a**) B6-2.5b; (**b**) B6-3.0b; (**c**) B6-4.0b (unit: mm).

**Figure 4:**
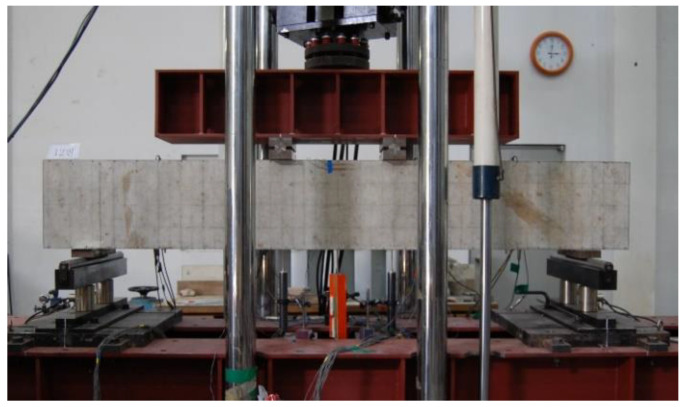
View of test setup of a specimen tested in the previous study [8,9].

As shown in Figure 3, the cross-sectional width, depth, and effective depth of the specimens were 200 mm, 400 mm, and 350 mm, respectively. The distance between the loading points was 500 mm, and four-point loading was applied to the specimens. To measure the curvature of the specimen and the strain of the materials, wire strain gauges were attached to the tension and compression reinforcing bars and to the concrete extreme compression fiber located at the mid-span of the specimen. As shown in Figure 4, a universal testing machine (UTM) with a capacity of 2000 kN was used for loading, and a LVDT were installed at the bottom of the specimens to measure the mid-span deflection. 

## 3. Evaluation of Shear Effect on Deflection of RC Beams 

### 3.1. Elastic Analysis

In this section, the shear effect on the beam deflection is evaluated using the virtual work method. The deflection of the beam varies according to the loading type and the boundary conditions. When the magnitude of the load is the same, the simple support rather than the fixed support, as well as the concentrated load closer to the mid-span rather than the uniformly distributed load, causes larger deflection. As shown in Figure 2, the deflection of the beam is affected by flexure and shear, and the total deflection $\Delta_{t}$ of the mid-span of the beam is calculated using the virtual work method as follows:

(1) $$\Delta_{t}=\Delta_{f}+\Delta_{s}=\int \frac{Mm}{EI}dx+\chi \int \frac{Vv}{GA}dx$$

where $\Delta_{f}$ and $\Delta_{s}$ are the deflections due to flexure and shear, respectively, M and V are the bending moment and shear force, respectively, m and v are the moment and shear force induced by virtual work, respectively, E is the elastic modulus, I is the moment of inertia, χ is the factor according to cross-sectional type (1.2 for rectangular), G is the shear modulus of elasticity (=E/2(1+ν)), ν is the Poisson’s ratio, and A is the cross-sectional area. The first and second terms of Equation (1) mean deflection due to flexure and shear, respectively. 

For a simply supported beam, the total deflection at the mid-span of the beam can be calculated using Equation (1) for the case of a four-point load and a uniformly distributed load as follows:

(2) $$\Delta_{t}=\frac{Pa}{48EI}(3l^{2}-4a)+\frac{\chi Pa}{2GA}\;(\mathrm{for}\;\mathrm{four-point}\;\mathrm{load})$$

(3) $$\Delta_{t}=\frac{5wl^{4}}{384EI}+\frac{\chi wl^{2}}{8GA}\;(\mathrm{for}\;\mathrm{uniform}\;\mathrm{load})$$

where P and w are the concentrated and uniform loads, respectively. The first term of Equations (2) and (3) is the deflection due to flexure, and the second term is the one due to shear. 

By substituting the characteristics of RC beams with a rectangular cross-section, that is, ν=0.16, $G=0.43E_{c}$, $E=E_{c}$, $I=bh^{3}/12$, A=bh, and d≈0.9h, into Equations (2) and (3), and by generalizing the deflection, the following equation is derived:

(4) $$\Delta_{t}=\Delta_{f}\left[1+C_{s}{\left(\frac{d}{l}\right)}^{2}\right]$$

where $C_{s}$ is the factor dependent on the loading type. $C_{s}$ is 3.4 for the central concentrated load and 2.8 for the uniformly distributed load. As shown in Equation (4), the effect of shear on the deflection in the elastic theory is proportional to the square of d/l. 

**Figure 5:**
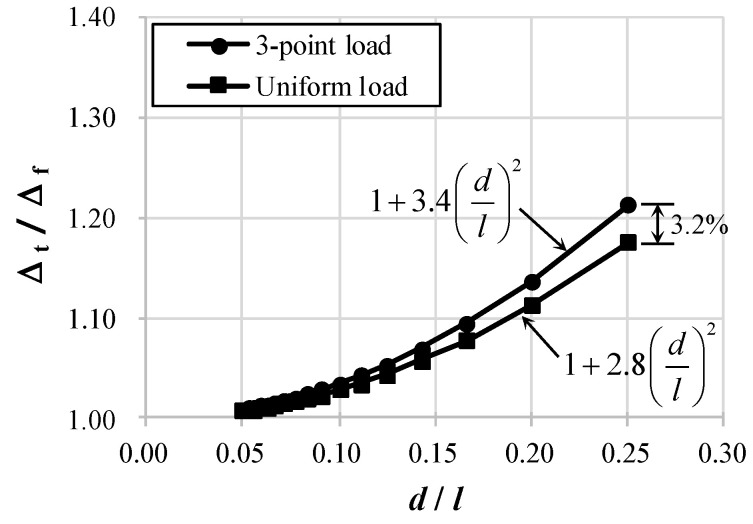
Calculation Results of Elastic Analysis using Equation (4).

Figure 5 shows the $\Delta_{t}/\Delta_{f}$ value of Equation (4) according to the change in d/l. $\Delta_{t}/\Delta_{f}$ is the ratio of the total deflection to the flexural deflection of the beam. As the ratio $\Delta_{t}/\Delta_{f}$ increases, the effect of shear on deflection increases. In the case of d/l≤0.1, there is little difference in the effect of shear by the load pattern. Even if d/l is increased to 0.25, as shown in Figure 5, the difference between two load patterns is only 3.2%. However, as d/l increases to 0.25, the $\Delta_{t}/\Delta_{f}$ ratio is approximately 1.2, confirming that the shear deflection is approximately 20% of the flexural deflection, where d/l = 0.25 corresponds to a/d = 2.0 for beams subjected to a central concentrated load. The ratio $\Delta_{t}/\Delta_{f}$ at d/l = 0.125 with a/d = 4.0 is approximately 1.05, meaning that the shear deflection is as small as approximately 5% of the flexural deflection. 

In general, the deflection of RC beams tends to be calculated by ignoring the effect of shear based on elastic theory. However, in recent design trends, the use of long-span RC beams with large d/l is increasing. Thus, it is very important to consider the amount of deflection due to shear. In addition, cracks, which are characteristics of RC structures, are not reflected in Equation (4) and may differ from the actual characteristics. In particular, shear cracks of RC members not only occur at an inclined angle but can also induce larger deflection because the deformation after cracking is concentrated in the cracks. In the next section, the experimental evaluation of the deflections due to flexure and shear and their comparison with the theoretical values are detailed.

### 3.2. Experimental Approach

Figure 2 shows the method used to measure the deflection due to flexure and shear. The mid-span deflection of the RC beam measured from the LVDT indicates the combined deflection of flexure and shear, as shown in Figure 1. On the other hand, the strains measured from the strain gauges attached to the mid-span of the RC beam is used to obtain the flexural deflection using the curvature of the section, moment of inertia, and elastic deflection equation. Table 2 indicates the experimental and analytical results of specimens at the first yield of tension reinforcement. The experimental results for deflection are $\Delta_{t,\exp.}$ measured from the LVDT installed at the mid-span of the beam specimens and $\Delta_{f,\exp.}$ obtained using the attached strain gauges at the mid-span of the specimens. The coefficient of variation (COV) in Table 2 is the standard deviation divided by the mean value of predicted results. 

The flexural deflection $\Delta_{f,\exp.}$ can be obtained using the following equations:

(5) $$\Delta_{f,\exp.}=\frac{M_{y}}{24E_{c}I_{e,exp.}}(3l^{2}-4a^{2})$$

(6) $$I_{e,\exp.}=\frac{M_{y}}{E_{c}\varphi_{\exp.}}$$

(7) $$\varphi_{\exp.}=\frac{\varepsilon_{c}+\varepsilon_{s}}{d}$$

where $M_{y}$ is the yield moment, $I_{e,exp.}$ is the effective moment of inertia obtained from the curvature relationship of the section at the position where deflection is considered, $\varphi_{exp.}$ is the curvature obtained from the attached strain gauges, $\varepsilon_{c}$ is the strain of the concrete extreme compression fiber, and $\varepsilon_{s}$ is the strain of tension reinforcement. 

Substituting Equation (6) into Equation (5), the following relationship can be obtained:

(8) $$\Delta_{f,exp.}=\frac{\varphi_{exp.}}{24E_{c}}(3l^{2}-4a^{2})$$

**Figure 6:**
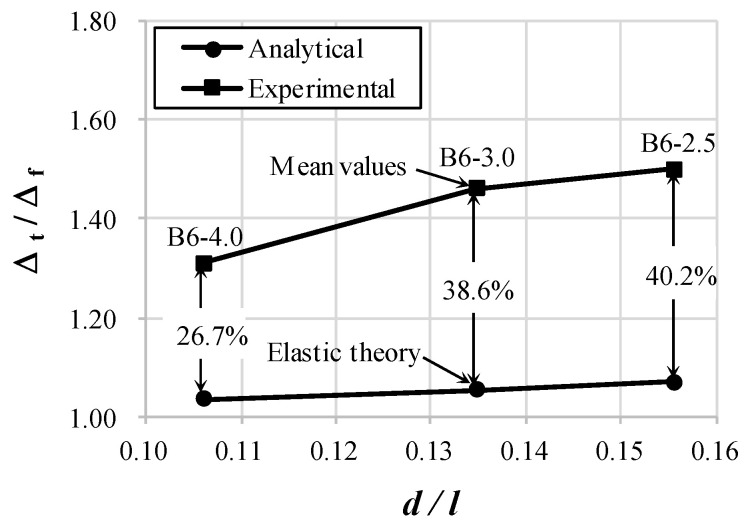
Comparison between experimental and elastic analytical results at flexural yield.

Equation (8) indicates that $\Delta_{f,exp.}$ is determined by the curvature obtained from the strain gauges depended on the flexural deformation. As shown in Table 2, as the shear span-to-depth ratio decreases from 4.0 to 2.5, the ratio of total deflection to flexural deflection increases from 1.31 to 1.49 on average. This implies that the shear deflection corresponds to 31% to 49% of the flexural deflection. In other words, as the shear span-to-depth ratio decreases, the effect of shear increases, and the difference between the total deflection and flexural deflection also increases. As shown in Figure 6, the shear effect measured in the experiment was 26.7–40.2% higher than that calculated by the virtual work method. This is because that the inclined cracks caused by shear in RC beams induced greater shear deformation. 

## 4. Prediction of Deflection of Flexure-Critical RC Beams 

### 4.1. ACI Provisions 

The ACI 318 committee recommends that the deflection of RC beams under service loads can be calculated using the effective moment of inertia considering crack characteristics. The effective moment of inertia $I_{e}$ is used to calculate the deflection by substituting it into the elastic deflection equation as shown in Equation (5). In the ACI 318 building code, the Branson equation was used up to ACI 318-14 [3]; however, the Bischoff equation was used in ACI 318-19 [7].

(9) $$I_{e,ACI}={\left(\frac{M_{cr}}{M_{a}}\right)}^{3}I_{g}+\left[1-{\left(\frac{M_{cr}}{M_{a}}\right)}^{3}\right]I_{cr}\;(\mathrm{for}\;\mathrm{ACI}\;318-14)$$

(10) $$I_{e,ACI}=\frac{I_{cr}}{1-{\left(\frac{(2/3)M_{cr}}{M_{a}}\right)}^{2}\left(1-\frac{I_{cr}}{I_{g}}\right)}\;(\mathrm{for}\;\mathrm{ACI}\;318-19)$$

Table 2 presents the analytical results on the deflection at flexural yield using Equations (9) and (10). As shown in Table 2, there is little difference between the analytical results of ACI 318-14 and ACI 318-19. This is because the difference between the two formulas occurs when the tension reinforcement ratio is less than 1% [5]. $\Delta_{y,ACI}$, the analytical result obtained by substituting $I_{e,ACI}$ of Equation (10) instead of $I_{e,\exp.}$ in Equation (5), significantly underestimated the LVDT deflection $\Delta_{t,\exp.}$ with an average of 1.42. Underestimating the real deflection is undesirable in terms of the serviceability of structures. 

The deflection using Equation (4) derived from the virtual work method and the LVDT deflection $\Delta_{t,\exp.}$ obtained from the experiment are compared in Table 2. As shown in Table 2, ACI 318-19 still considerably underestimated the experimental results with an average of 1.35. In contrast, $\Delta_{y,ACI}$ calculated using ACI 318-19 almost coincided with $\Delta_{f,\exp.}$ experimentally obtained using Equations (5)–(7) with an average of 1.00. This implies that the analytical method using the elastic deflection equation and the effective moment of inertia proposed by the ACI 318 code can predict well the flexural deflection of RC beams but not the total deflection, considering both the flexural and shear effects. The deflection due to shear deformation of RC beams is larger than that in the elastic theory, whereas studies considering the shear effect are insufficient. Therefore, a new evaluation method considering the effect of shear on the deflection is required. 

### 4.2. Calculation Method Considering Shear Effect on Deflection of RC Beams 

The total deflection of RC beams can be expressed by the following equation which multiplies the flexural deflection by an incremental factor:

(11) $$\Delta_{t}=\alpha_{s}\Delta_{f}$$

where $\alpha_{s}$ is the incremental factor considering shear effect. The $\Delta_{f}$ can be obtained using the method recommended by ACI 318-19. 

**Figure 7:**
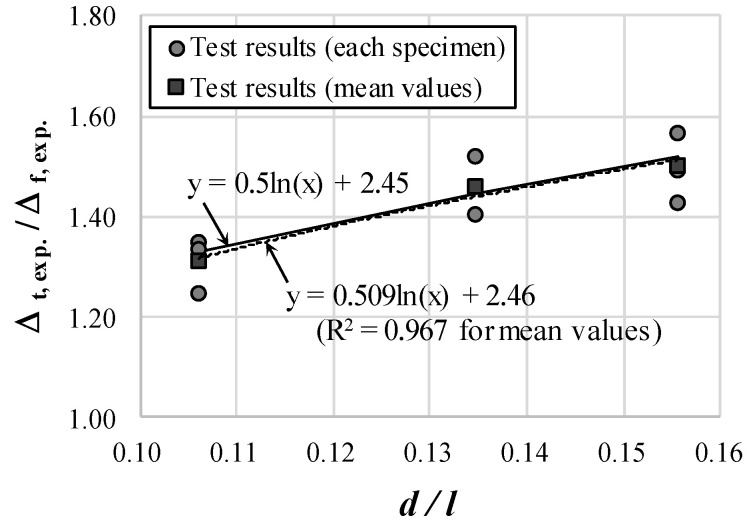
Regression analysis of experimental results.

Figure 7 shows the ratio $\Delta_{t,exp.}/\Delta_{f,exp.}$ according to d/l presented in Table 2. The circular mark indicates the test result for each of the nine specimens, and the square mark indicates the average value for each series of specimens. Furthermore, $\Delta_{t,exp.}/\Delta_{f,exp.}$ is the ratio of the total deflection to the flexural deflection of RC beams. This ratio has the same meaning as $\alpha_{s}$ in Equation (11) and is an incremental value of deflection due to the effect of shear. The results of the regression analysis using the least-squares method for the mean values of the experimental results for each series are shown as a dotted line in Figure 7. Considering practicality, the deflection incremental factor $\alpha_{s}$ can be proposed as follows:

(12) $$\alpha_{s}=0.5\ln\;\left(\frac{d}{l}\right)+2.45$$

where $1.0\leq \alpha_{s}\leq 1.65$.

### 4.3. Verification of Proposed Method

In this study, a total of 60 existing experimental results [9,11,12,13,14,15,16,17,18,19,20,21,22,23,24] were collected from the literature to verify the proposed method using the deflection incremental factor $\alpha_{s}$. Table 3 shows the details of the collected data and the comparison results between the experimental and analytical results. The collected specimens were simply supported beams subjected to four-point load and failed in flexure before the shear reinforcement yielded. The beams had a concrete compressive strength of 20.3–58.0 MPa, a beam width of 140–400 mm, a beam height of 250–600 mm, a shear span-to-depth ratio of 2.3–7.1, a d/l of 0.066–0.156, a yield strength of the tension steel bar of 379.7–543.4 MPa, a tension reinforcement ratio of 0.004 to 0.03 (=0.15–0.78$\rho_{b}$), and $\rho_{b}$ is the balanced reinforcement ratio of a singly reinforced section. 

**Figure 8:**
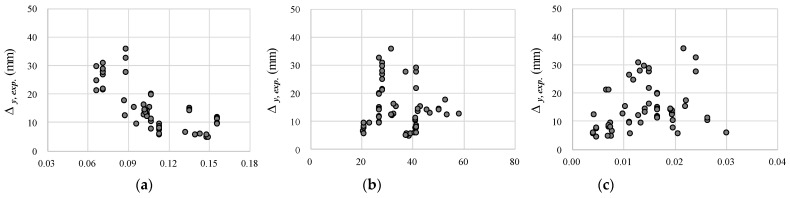
Effect of test variables on deflection at flexural yield: (**a**) *d/l* ratio; (**b**) concrete compressive strength; (**c**) tension steel ratio.

Figure 8 shows the effect of the test variables on the deflection at the yield moment of the collected beams. Figure 8a shows the effect of d/l on the experimental results. As d/l increases, the tendency to decrease the deflection at flexural yield is clear. However, the characteristics of the concrete and tension reinforcement have little effect on the experimental results, as shown in Figure 8b,c. As shown in Equations (2)–(4), d/l directly affects the deflection, whereas the material properties affect both the load capacity and moment of inertia. In other words, it is not easy to directly evaluate the effect of material properties on deflection from the experimental results with various test variables. The effect of material properties on the deflection of RC beams requires further study. 

The load $P_{y,ACI}$ in Table 3 was calculated using the theory of the sectional analysis of RC beams [25] and ACI 318-19 [7]. As shown in Table 3, the analytical results for the yield load of 60 specimens were in good agreement with the experimental results with an average of 1.01 and a COV of 12.6%. In contrast, the predicted result of ACI 318-19 for the yield deflection $\Delta_{y,\exp.}$ was 1.33 on average, which considerably underestimated the experimental results. This is similar to the prediction results for the previous experimental results in Section 4.1. 

**Figure 9:**
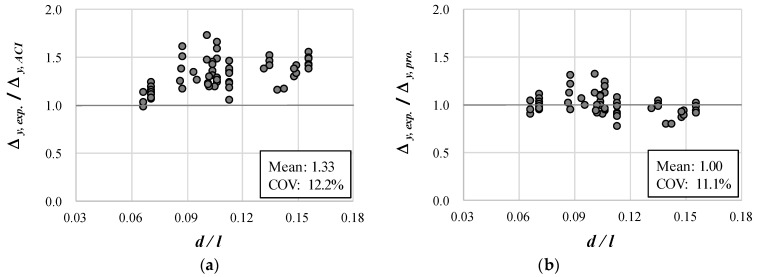
Prediction results of analytical methods on deflection at flexural yield: (**a**) ACI 318-19; (**b**) Proposed method.

In particular, Figure 9a, which shows the $\Delta_{y,exp.}/\Delta_{y,ACI}$ for yield deflection, indicates the ACI 318-19 method underestimates the experimental results more as d/l increases. This is because the ACI 318-19 method considers only the deflection due to flexure. Figure 9b shows the results of predicting the experimental results using Equations (11) and (12) proposed in this study. Figure 9b indicates that, when the proposed method is used, the collected experimental results can be predicted relatively well without being greatly affected by d/l. In particular, as shown in Table 3, the analytical results using the proposed method accurately predicted the experimental results for yield deflection with an average of 1.0 and a COV of 11.1%. Therefore, the proposed method can be used to improve the existing calculation method that considers only the effect of flexure, including the current ACI building code. 

## 5. Conclusions

In this paper, a method for calculating the deflection of flexure-critical RC beams considering the effect of shear was proposed. The deflection incremental coefficient considering shear effect was proposed based on an analysis of the experimental results. The following conclusions were drawn by comparing the experimental and analytical results:

1. The shear deflection of RC beams calculated from the elastic bending theory underestimated the real shear deflection by up to approximately 40% as d/l increased. This is because the crack characteristics of the RC structure were not reflected in the elastic bending theory. An analytical method that considers the effect of shear on deflection should be used to reasonably predict the deflection of RC beams;
2. The ACI 318-19, which calculates the deflection using the effective moment of inertia, was found to significantly underestimate the real total deflection of RC beams. Furthermore, the tendency to underestimate increased as d/l increased. Meanwhile, the deflection calculated using ACI 318-19 was very similar to the flexural deflection of RC beams measured from strain gauges with an average of 1.0;
3. In this study, the deflection incremental coefficient considering shear effect and a method for calculating the deflection of RC beams were devised. The proposed deflection incremental coefficient was applied to the flexural deflection calculated using ACI 318-19 to evaluate the total deflection of the RC beams. By comparing the experimental and analytical results, the proposed method using the deflection incremental coefficient predicted the real total deflection of RC beams well with an average of 1.0 and a COV of 11.1%.

## Figures and Tables

**Table 1:** Details of Specimens and Experimental Results at Flexural Yield [8,9].

Specimens	*a/d* (*d/l*)	Tension Rebar (Comp.)	Shear Rebar	$\frac{V_{shear}}{V_{flexure}}$	$P_{y,exp.}$ (kN)	$\Delta_{t,\exp.}$ (mm)
B6-2.5a	2.5 (0.156)	3-D22 (2-D16)	D10@95 mm	1.1	451.6	11.9
B6-2.5b			D10@70 mm	1.4	446.8	11.5
B6-2.5c			D10@55 mm	1.7	467.0	11.4
B6-3.0a	3.0 (0.135)		D10@120 mm	1.1	382.2	15.2
B6-3.0b			D10@90 mm	1.4	373.5	14.6
B6-3.0c			D10@70 mm	1.7	372.3	14.2
B6-4.0a	4.0 (0.106)		D10@180 mm	1.1	275.1	19.8
B6-4.0b			D10@130 mm	1.4	268.7	20.1
B6-4.0c			D10@100 mm	1.7	274.1	19.9

Note: *a*—shear span, *d*—effective depth, *l*—clear span of specimens, $V_{shear}$ and $V_{flexure}$—nominal shear and flexural strengths calculated by ACI 318-14, respectively, $P_{y,exp.}$—applied load at flexural yield, and $\Delta_{t,\exp.}$—total deflection measured from LVDT installed at the mid-span of the beam specimens.

**Table 2:** Comparison of Experimental and Analytical Results at Flexural Yield.

Specimens	Experimental Results		ACI 318-14				ACI 318-19		
	$\Delta_{t,\exp.}$ (mm)	$\Delta_{f,\exp.}$ (mm)	$\frac{\Delta_{t,\exp.}}{\Delta_{f,\exp.}}$	$\frac{\Delta_{t,\exp.}}{\Delta_{y,ACI}}$	$\frac{\Delta_{t,\exp.}}{\Delta_{t,Eq.(4)}}$	$\frac{\Delta_{f,\exp.}}{\Delta_{y,ACI}}$	$\frac{\Delta_{t,\exp.}}{\Delta_{y,ACI}}$	$\frac{\Delta_{t,\exp.}}{\Delta_{t,Eq.(4)}}$	$\frac{\Delta_{f,\exp.}}{\Delta_{y,ACI}}$
B6-2.5a	11.9	7.6	1.57	1.55	1.45	0.99	1.56	1.46	1.00
B6-2.5b	11.5	7.7	1.49	1.50	1.40	1.00	1.51	1.41	1.01
B6-2.5c	11.4	8.0	1.43	1.49	1.39	1.04	1.49	1.40	1.05
B6-3.0a	15.2	10.0	1.52	1.51	1.44	1.00	1.52	1.45	1.00
B6-3.0b	14.6	10.0	1.46	1.45	1.38	1.00	1.46	1.39	1.00
B6-3.0c	14.2	10.1	1.41	1.41	1.34	1.01	1.42	1.35	1.01
B6-4.0a	19.8	14.7	1.35	1.26	1.22	0.94	1.27	1.23	0.94
B6-4.0b	20.1	16.1	1.25	1.28	1.24	1.03	1.29	1.24	1.03
B6-4.0c	19.9	14.9	1.34	1.27	1.23	0.95	1.27	1.23	0.95
Mean			1.42	1.41	1.34	0.99	1.42	1.35	1.00
COV(%)			7.0	8.2	6.9	3.4	8.2	6.9	3.4

Note: $\Delta_{f,exp.}$ —flexural deflection calculated from the curvature relationships using the attached strain gauges, $\Delta_{y,ACI}$ —yield deflection calculated using ACI building code, and $\Delta_{t,Eq.(4)}$ —total deflection calculated using Equation (4).

**Table 3:** Comparison of Observed and Predicted Results of Flexure-Critical RC Beams Reported in the Literature.

Ref.	Specimens	$f_{c}^{\prime }$ (MPa)	*b* (mm)	*h* (mm)	a/d	d/l	$P_{y,\exp.}$ (kN)	$\Delta_{y,\exp.}$ (mm)	$\frac{P_{y,\exp.}}{P_{y,ACI}}$	$\frac{\Delta_{y,\exp.}}{\Delta_{y,ACI}}$	$\frac{\Delta_{y,\exp.}}{\Delta_{y,pro.}}$
11	4B4-0.5(0)	41.0	140	260	4.0	0.106	106.4	7.9	0.98	1.25	0.94
	4B4-0.5(10)	41.0	140	260	4.0	0.106	113.1	10.4	1.04	1.66	1.25
	4B4-0.7(10)	41.0	140	260	4.0	0.106	143.8	10.6	0.97	1.49	1.12
	4B4-0.7(5)	41.0	140	260	4.0	0.106	146.1	11.2	0.98	1.59	1.20
12	R1	38.2	200	300	2.3	0.149	111.0	4.7	1.25	1.42	0.94
	R2	37.5	200	300	2.3	0.147	185.0	5.0	1.33	1.31	0.88
	R3	37.3	200	300	2.5	0.139	340.0	5.7	1.10	1.17	0.80
	R4	37.0	200	300	2.3	0.149	189.0	5.0	1.44	1.34	0.90
	R5	39.1	200	300	2.3	0.147	308.0	5.7	1.50	1.39	0.93
	R6	40.7	200	300	2.4	0.142	495.0	6.1	1.07	1.17	0.80
13	A211	42.8	250	400	3.4	0.105	440.5	15.5	0.94	1.20	0.91
14	1	33.1	150	300	3.8	0.094	100.3	15.4	1.04	1.35	1.07
	2	52.5	150	300	4.1	0.086	170.3	17.6	0.99	1.26	1.03
15	BG0	46.7	200	350	4.0	0.103	222.3	13.1	0.98	1.36	1.03
	BG30	58.0	200	350	4.0	0.103	222.2	12.8	0.97	1.36	1.03
	BG50	53.1	200	350	4.0	0.103	216.8	12.5	0.95	1.31	1.00
	BG70	45.2	200	350	4.0	0.103	241.5	14.1	1.06	1.46	1.11
16	AN24-0.3	32.4	200	300	4.0	0.101	112.3	12.7	1.03	1.48	1.13
	AN24-0.5	32.4	200	300	4.0	0.101	168.9	16.2	1.04	1.73	1.33
17	F-AN	31.7	200	350	4.0	0.103	149.2	12.2	1.01	1.43	1.09
9	B6-2.5a	26.8	200	400	2.5	0.156	451.6	11.9	1.01	1.56	1.03
	B6-2.5b	26.8	200	400	2.5	0.156	446.8	11.5	1.00	1.51	0.99
	B6-2.5c	26.8	200	400	2.5	0.156	467	11.4	1.05	1.49	0.98
	B6-3.0a	26.8	200	400	3.0	0.135	382.2	15.2	1.04	1.52	1.05
	B6-3.0b	26.8	200	400	3.0	0.135	373.5	14.6	1.00	1.46	1.01
	B6-3.0c	26.8	200	400	3.0	0.135	372.3	14.2	1.00	1.42	0.98
	B6-4.0a	26.8	200	400	4.0	0.106	275.1	19.8	0.99	1.27	0.95
	B6-4.0b	26.8	200	400	4.0	0.106	268.7	20.1	0.96	1.29	0.97
	B6-4.0c	26.8	200	400	4.0	0.106	274.1	19.9	0.98	1.27	0.96
	B3-2.5a	26.8	200	400	2.5	0.156	285.3	9.9	0.94	1.44	0.95
	B3-2.5b	26.8	200	400	2.5	0.156	299.7	9.8	0.99	1.42	0.94
	B3-2.5c	26.8	200	400	2.5	0.156	296.1	9.5	0.97	1.39	0.91
18	AN	20.3	300	300	3.2	0.132	173.3	6.6	1.10	1.39	0.97
19	BFO1	50.0	200	350	4.1	0.102	183.0	14.5	0.92	1.31	1.00
	BFO2	50.0	200	350	4.1	0.101	252.0	14.2	1.00	1.24	0.95
	BFO3	41.7	200	350	4.1	0.102	190.0	13.5	0.96	1.19	0.91
	BFO4	41.7	200	350	4.1	0.101	244.0	14.4	0.97	1.23	0.94
20	B-R0.75-A0	37.0	400	600	5.1	0.088	649.1	27.6	0.88	1.17	0.95
	BFS4-A0	26.8	400	600	5.1	0.088	581.0	32.6	0.91	1.51	1.23
	BFS5-A0	31.5	400	600	5.1	0.088	619.8	35.9	0.99	1.62	1.32
21	G-X13	28.0	250	350	4.5	0.070	86.3	21.4	0.95	1.07	0.96
	G-X16	28.0	250	350	4.5	0.070	173.1	31.0	0.93	1.25	1.11
	G-X19	28.0	250	350	4.5	0.070	130.1	26.7	0.86	1.20	1.07
	G-X25	28.0	250	350	4.5	0.070	155.3	27.9	0.85	1.16	1.03
	G-Y13	28.0	250	350	4.8	0.066	77.2	21.2	0.91	0.99	0.91
	G-Y19	28.0	250	350	4.8	0.066	113.2	24.9	0.81	1.03	0.95
	G-Y25	28.0	250	350	4.8	0.066	142.7	29.8	0.84	1.14	1.05
	SN-0	41.1	250	350	7.1	0.070	123.0	21.8	0.87	1.14	1.01
	SN-1	41.1	250	350	4.5	0.070	187.0	27.6	0.84	1.11	0.99
	SN-2	41.1	250	350	3.2	0.070	269.0	29.0	0.86	1.09	0.97
22	N-10-3	20.6	200	300	3.7	0.113	47.6	5.8	1.02	1.06	0.78
	N-13-2	20.6	200	300	3.7	0.113	63.8	7.6	1.09	1.25	0.92
	N-13-3	20.6	200	300	3.7	0.113	92.1	8.2	1.07	1.24	0.91
	N-16-2	20.6	200	300	3.7	0.113	95.1	9.5	1.09	1.46	1.08
	H-10-3	41.3	200	300	3.7	0.113	48.2	5.9	1.02	1.19	0.88
	H-13-2	41.3	200	300	3.7	0.113	65.2	7.7	1.10	1.37	1.01
	H-13-3	41.3	200	300	3.7	0.113	95.0	8.6	1.09	1.39	1.02
	H-16-2	41.3	200	300	3.7	0.113	96.5	8.1	1.09	1.33	0.98
23	Control	31.3	200	300	4.0	0.087	46.7	12.5	1.10	1.39	1.13
24	F0	22.8	150	250	3.5	0.095	69.4	9.4	0.88	1.27	0.99
Mean									1.01	1.33	1.00
COV									12.6%	12.2%	11.1%

Note: $f_{c}^{\prime }$—compressive strength of concrete, *h*—depth of RC beams, $\Delta_{y,\exp.}$—experimental total deflection at flexural yield, $P_{y,ACI}$—yield load calculated using ACI code, and $\Delta_{y,pro.}$—total deflection obtained using proposed method.

## Data Availability

Not applicable.
